# Collective Expert Perspectives on the Use of Safinamide as Adjunctive Therapy for Parkinson's Disease: Online-Based Delphi Survey

**DOI:** 10.1155/2022/3203212

**Published:** 2022-07-15

**Authors:** Atsushi Takeda, Yoshio Tsuboi, Masahiro Nomoto, Hideki Mochizuki, Nobutaka Hattori

**Affiliations:** ^1^Department of Neurology, National Hospital Organization, Sendai Nishitaga Hospital, 2-11-11 Kagitorihoncho, Sendai Taihaku-ku, Miyagi 982-8555, Japan; ^2^Department of Cognitive and Motor Aging, Tohoku University Graduate School of Medicine, 2-1 Seiryo-machi, Sendai Aoba-ku, Miyagi 980-8575, Japan; ^3^Department of Neurology, Fukuoka University, 7-45-1 Nanakuma, Jonan-ku, Fukuoka 814-0180, Japan; ^4^Department of Neurology, Saiseikai Imabari Center for Health and Welfare, 7-61 Kitamura, Imabari, Ehime 799-1592, Japan; ^5^Department of Neurology, Osaka University Graduate School of Medicine, 2-15 Yamadaoka, Suita, Osaka 565-0871, Japan; ^6^Department of Neurology, Juntendo University School of Medicine, 2-1-1 Bunkyo-ku, Tokyo 113-8421, Japan

## Abstract

**Background:**

Safinamide is a selective, reversible monoamine oxidase-B inhibitor with a sodium channel inhibitory effect. Published clinical evidence supports safinamide as an effective therapy for Parkinson's disease (PD) with wearing-off. However, to date, no consensus recommendations have been available to guide physicians in Asia on the optimal use of safinamide in clinical practice. To summarize opinions on the optimal patient profile and methods of using safinamide in common clinical scenarios, Japanese movement disorder specialists with expertise in PD investigated the perspectives of neurologists and neurosurgeons.

**Methods:**

The Delphi panel approach was used to summarize the opinions of panelists. The panel comprised doctors from Japan with extensive clinical practice experience in the use of safinamide (*n* = 46 at the final round). The consensus was defined as 80% or more agreement between panelists for each scenario at the final round.

**Results:**

There was a high level of agreement that patients with the following symptoms are suitable for safinamide treatment such as bradykinesia (100%), rigidity (95.7%), and/or gait disorder (89.1%) based on motor symptoms and PD-related pain (97.8%) and/or depression or apathy (93.5%) based on non-motor symptoms. Morning-off (95.7%), but not dyskinesia (71.7%), also reached consensus. The use of high-dose safinamide (100 mg/day) was recommended when the improvement in PD symptoms is insufficient and increasing the doses of other anti-PD medications is difficult (97.8%) or when the abovementioned non-motor symptoms adversely affect daily life (93.5%).

**Conclusions:**

This report provides expert perspectives on the use of safinamide for a wide range of clinical scenarios in Japan.

## 1. Introduction

The pharmacological management of Parkinson's disease (PD) has improved, but motor complications, including wearing-off and dyskinesia, can become problematic as the disease progresses. From the patient's perspective, non-motor complications, including pain and mood disorders, are also common complaints [1].

Safinamide is a selective, reversible monoamine oxidase (MAO)-B inhibitor that also inhibits glutamate release by blocking sodium channels [2, 3]. Safinamide was first approved in 2015 in the European Union, with subsequent approvals obtained in the United States (2017) and Japan (2019) as an add-on therapy to levodopa for patients with PD who are experiencing wearing-off symptoms [4].

The efficacy and safety of safinamide (50 and 100 mg/day) have been demonstrated in multiple placebo-controlled, double-blind studies and meta-analyses in approved patient populations [4]. In addition to its effects on motor symptoms, post hoc analyses and clinical research studies have shown safinamide to have beneficial effects on non-motor symptoms related to pain and mood [5–7]. However, because of regional differences in clinical practice and differences in the availability of medications between Asia (including Japan) and Europe or North America, there is no global consensus on the most appropriate use of safinamide. Furthermore, there are no clinical guidelines established in Japan or elsewhere.

Evidence from clinical practice and real-world data on safinamide use are limited, and there are many unknown factors regarding the most appropriate patient group for this treatment, as well as adverse event (AE) management. Investigating optimal treatment strategies among various clinical scenarios is useful for physicians prescribing safinamide for the first time and for experienced physicians who need to manage AEs. This study used an online survey to gather expert consensus on the optimal use of safinamide from specialists with experience in prescribing safinamide for the treatment of PD.

## 2. Materials and Methods

### 2.1. Study Design

This study was performed by a steering committee consisting of this article's five authors, all of whom are PD specialists in Japan. Committee meetings were organized by Eisai Co., Ltd., the manufacturer and distributor of safinamide in Asia. Two meetings were held to discuss the design of this study and the interpretation of the results.

This study adopted the Delphi approach for consensus formation using an online questionnaire survey [8]. The online survey was conducted among a panel of Japanese doctors (>290,000 doctors), provided by m3.com. Answers were anonymized, and each panel member provided their answers independently without discussing with other panel members. The panel accessed the questionnaire via a website and answered after providing consent to participate in this study. The use of Secure Sockets Layer, a technology that encrypts communications on the Internet, eliminates the risks of being intercepted and tampered with by third parties.

The Medical Corporation Takahashi Clinic Ethics Committee approved the protocol for this study in February 2021 (UMIN000043283). All study procedures were performed after approval.

### 2.2. Delphi Rounds and Questionnaires

**Figure 1:**
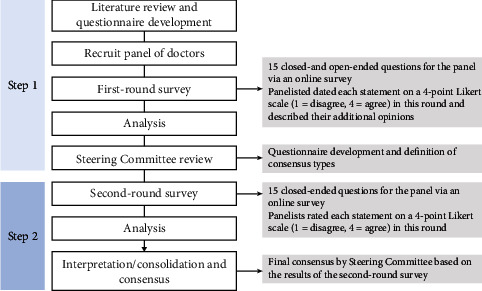
Delphi review process. Expert opinions and consensus recommendation process. The development process was based on the Delphi method for conceptualizing, designing, and carrying out the appropriate procedures for the diagnosis and treatment of Parkinson's disease. The method consists of a Delphi approach, in which a panel of Japanese Parkinson's disease experts assessed the appropriateness of clinical decisions in an iterative manner.

The panelists rated each statement on a 4-point Likert scale (1 to 4) in a two-round Delphi process (Figure 1). A score of “1” on the scale indicated “disagree,” “2” indicated “moderately disagree,” “3” indicated “moderately agree,” and “4” indicated “agree.”

The first-round questionnaire (conducted in February 2021) consisted of both closed-ended (Likert scale survey questions) and open-ended questions. The panelists rated each statement from 1 to 4, with space for free-description answers in which they could provide an alternative or additional answer. The purpose of the first-round questionnaire was twofold: first, it was designed to examine the appropriateness of available alternatives in the second-round questionnaire; and second, it was designed to develop alternatives for inclusion in the second-round questionnaire. The second-round questionnaire (conducted from May to June 2021) consisted only of close-ended questions with the partially modified first-round questionnaire based on the results of open-ended questions. The panelists rated each statement using the same scale as the first round. In addition, they were able to see the response rate for all of the results of the first round of questions.

The second-round results were analyzed quantitatively. The level of consensus was classified into three categories based on previous studies [8, 9]: agreement (Likert scale = 3 or 4) or disagreement (Likert scale = 1 or 2) of ≥80% was classified as “consensus,” agreement or disagreement of 60%–79% was classified as “nearing consensus,” and other outcomes were classified as “no consensus.”

### 2.3. Survey Participants

Participants in this study were selected using the inclusion and exclusion criteria at the time of providing consent for participation. In the first-round survey, doctors had to fully understand the contents of the study and provide voluntary consent via the website, be ≥30 years of age with ≥5 years of experience treating PD in clinical practice, had to have provided continuous clinical care for ≥10 patients with PD, and had to have prescribed safinamide to at least one patient. Participants were not allowed to submit multiple applications. Doctors who could not provide questionnaire answers themselves and did not thoroughly answer all questions were excluded. Participants of the second round were limited to those who were included in the first-round survey. Additionally, to be included in the second round, doctors were required to have experience prescribing safinamide to at least five patients with a ≥3-month prescription period for each patient. To improve the reliability of the data, stricter eligibility criteria (i.e., experience of safinamide use) were applied to the second-round questionnaire.

### 2.4. Literature Review

A literature search was performed to prepare the survey. A search for “safinamide” in PubMed and the “Ichushi” Japanese database of medical literature was conducted, targeting online-published articles up to May 2021. Clinical research studies, case reports, post hoc analyses, and meta-analyses were included; review papers and non-clinical studies were excluded. From the 223 articles found in the databases, 50 articles that met the selection criteria were thoroughly checked and categorized into 1 of 5 evidence levels based on the Oxford Centre for Evidence-Based Medicine (Supplementary Table 1, Supplementary Table 2).

## 3. Results

### 3.1. Participant Demographics

In the first round, 150 survey responses were collected from eligible panelists. Forty-six of the original 150 panelists were eligible for the second round; the mean age was 52.0 years, 93.5% were male, 80.4% were neurologists, and 19.6% were neurosurgeons. The mean duration of experience treating PD was 22.9 years, and the mean number of patients prescribed safinamide was 14.0 (Supplementary Table 3).

### 3.2. Optimal Patient Profiles

**Figure 2:**
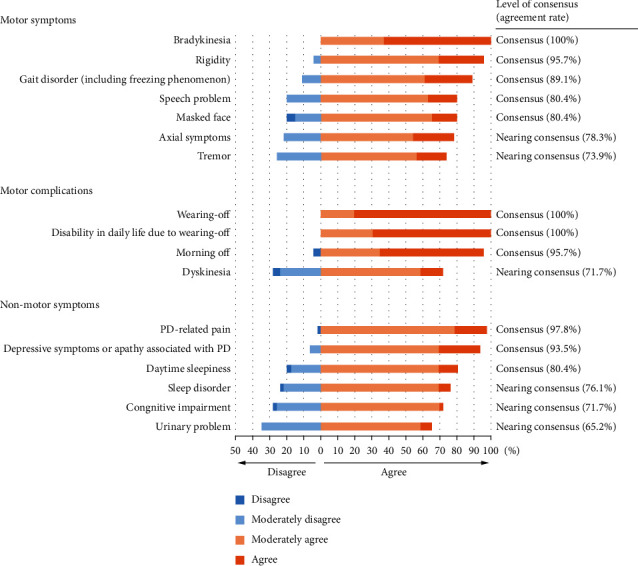
Summary of the optimal patient type with wearing-off for safinamide. Motor symptoms (Q1): optimal patient based on motor symptoms related to wearing-off; motor complications (Q2): optimal patient type based on motor complications; non-motor symptoms (Q9): optimal patient type based on non-motor symptoms associated with wearing-off. PD : Parkinson's disease.

The motor symptoms that achieved a consensus from the panel were bradykinesia, rigidity, gait disorder, speech problem, and masked face. For motor complications, wearing-off and resulting compromised activities of daily living (ADL) and morning-off achieved consensus. The non-motor symptoms that achieved a consensus were PD-related pain, depressive symptoms or apathy associated with PD, and daytime sleepiness (Figure 2; Q1, Q2, and Q9).

For initiation of safinamide, a complete (100%) agreement was reached on prior treatment condition, where patients were required to have experienced wearing-off with a levodopa-containing product used 4-5 times daily and have insufficient symptom improvement. All alternative scenarios for safinamide use in patients with wearing-off achieved a consensus (Supplementary Table 4; Q3 and q15).

The most highly agreed-upon condition for safinamide use regarding past use of a MAO-B inhibitor (except for safinamide) was MAO-B inhibitor naïve (97.8%), followed by the insufficient effect of another MAO-B inhibitor (95.7%), and AE related to another MAO-B inhibitor (91.3%) (Table 1; Q4).

### 3.3. Treatment Methods

**Figure 3:**
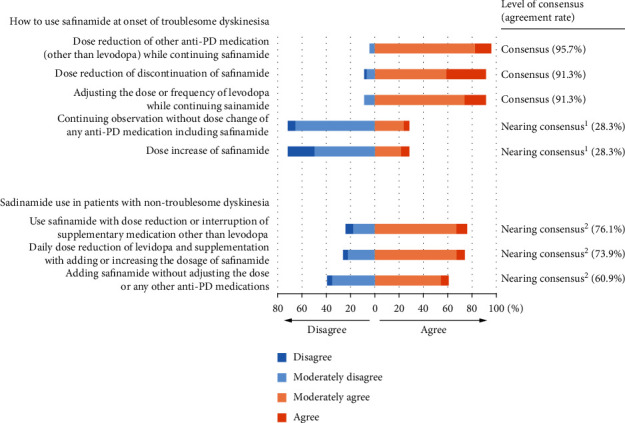
Expert opinions for actions to take in case of dyskinesia and safinamide use. Troublesome dyskinesia (Q5): action taken for troublesome dyskinesia during safinamide use; non-troublesome dyskinesia (Q6): action taken in using safinamide in patients with non-troublesome dyskinesia. ^1^Nearing consensus of disagreement. ^2^Nearing consensus of agreement. PD : Parkinson's disease.

The three possible actions in the case of troublesome dyskinesia achieved a consensus, but no scenarios concerning safinamide use in patients with non-troublesome dyskinesia achieved a consensus (Figure 3; Q5 and 6).

The three scenarios that supported increasing the dose of safinamide to 100 mg/day achieved a consensus of agreement, while a safinamide dose increase at the appearance of dyskinesia did not achieve a consensus. Three other scenarios achieved a consensus regarding dose reduction or discontinuation (Table 1; Q10 and Q11). The three scenarios regarding treatment for whom safinamide should be administered after carefully balancing risks and benefits achieved a consensus of agreement, while administration to patients with depressive symptoms or patients engaged in high-risk work, including car driving or operating machinery, did not achieve a consensus (Table 1; Q7). All scenarios concerning the use of safinamide in elderly patients achieved a consensus (Table 1; Q8).

Regarding the timing to evaluate the efficacy of safinamide, 2-3 months after the start of safinamide treatment achieved a higher consensus (87.0%) than 1 month after the start of safinamide (13.0%). A higher consensus on the timing of discontinuation or dose reduction was reached for 2-3 months after the onset of dyskinesia (91.3%) and immediately or within 1 month after the onset of other AEs (including hallucination and sleepiness) (89.1%) (Supplementary Table 4; Q12 and Q13).

Regarding the timing of administration, 39.1% of doctors indicated that there was no relationship between the effect of safinamide and the timing of administration. At least 80% of doctors answered that safinamide administration after dinner is appropriate if symptoms occur during the night. At least 80% of doctors answered that safinamide administration before bed was appropriate for early morning-off symptoms, and administration after breakfast was appropriate for daytime symptoms (Supplementary Table 4; Q14).

## 4. Discussion

There was strong agreement that patients with bradykinesia, rigidity, and/or gait disturbance are suitable for safinamide treatment. These results were consistent with a previous post hoc study in which safinamide improved bradykinesia, rigidity, and gait disturbance [10, 11]. Efficacy in patients with morning-off has also been shown in the results of the SETTLE study and a large observational study in Europe (SYNAPSES study) [12, 13], supporting our survey results. Dyskinesia reached an agreement nearing consensus, but in the 018 study, the efficacy of safinamide for dyskinesia was not confirmed [14]; as such, further confirmatory studies are required.

Regarding non-motor symptoms, PD-related pain, depression, and apathy obtained high agreement, followed by daytime sleepiness. Post hoc analyses of clinical studies indicated improved PD-related pain and depressive symptoms with safinamide [5–7]. Regarding pain and depression, improvements from baseline in King's Pain Scale for PD in an interventional study and improvement in Beck Depression Inventory II were reported [15]. Regarding daytime sleepiness, improvement in the Epworth Sleepiness Scale was reported in a retrospective cohort study [16], and safinamide was shown to improve sleep and daytime sleepiness in PD patients in the open-label SAFINONMOTOR study [17]. Reduced sleepiness has also been reported with the use of rasagiline [18] and selegiline [19]. Our results suggest the possibility that safinamide may have a lower risk of inducing somnolence than dopamine agonists.

Our results show that physicians believe that safinamide treatment can be used across a broad range of patients with wearing-off. In a post hoc analysis of a phase 3 study, the effect of safinamide was not influenced by the presence or absence of prior concomitant anti-PD medication in patients taking levodopa [10]. However, evidence regarding the efficacy and safety of concomitant use with device therapy (deep brain stimulation and levodopa continuous intestinal gel) is limited and needs further investigation because this combination was excluded from previous clinical studies.

In this study, doctors considered safinamide a treatment option for MAO-B inhibitor-naïve patients and patients in whom the tolerability or efficacy of other MAO-B inhibitors is insufficient. This result may be affected by the reversible action of safinamide, which is different from current non-reversible MAO-B inhibitors and by the non-dopaminergic effect of safinamide. Regarding efficacy, more than 1-hour improvement of average daily ON time compared with placebo was shown in a clinical study in Japan, in both 50 and 100 mg/day groups [20]. Other cohort studies suggested the possibility of a more substantial improvement with safinamide 100 mg/day on wearing-off than other MAO-B inhibitors [21]. Furthermore, it has been reported in multiple studies that the concomitant use of tyramine with both a clinical dose and an overdose of safinamide did not increase blood pressure (i.e., the tyramine response) [22, 23]. Based on these results, the authors consider that safinamide may have a negligible influence on blood pressure variability and a low risk of orthostatic hypotension. In Japan, when switching from MAO-B inhibitors, a wash-out period is required, and concomitant use of multiple MAO-B inhibitors is contraindicated, although an overnight switch from rasagiline to safinamide was reported as safe [24].

When troublesome dyskinesia occurs, dose reduction or discontinuation of safinamide and adjustment of other medications while continuing safinamide reached a consensus. This finding may reflect the Japanese PD guideline issued in 2018 before the market launch of safinamide [25]. The guideline recommended that when troublesome dyskinesia occurs, MAO-B inhibitors should be reduced or discontinued. Decisions regarding the continuation or discontinuation of safinamide are also based on the balancing of troublesome dyskinesia and motor symptoms because dose reduction may worsen motor symptoms. Conversely, a dose increase in safinamide reached a near consensus of disagreement in this scenario. As mentioned above, the efficacy of safinamide (50 or 100 mg/day) for dyskinesia was not confirmed in the 018 study. Post hoc analysis in the subgroup of patients who had moderate or severe dyskinesia indicated the efficacy of safinamide 100 mg [14]. However, clear conclusions regarding safinamide's effect on dyskinesia have not been obtained.

There is no established recommendation for the use of safinamide in patients who already have non-troublesome dyskinesia, and our study did not reach a consensus on the use of safinamide in these patients. When the benefit of efficacy exceeds the risk of worsening dyskinesia with safinamide use, using safinamide should be considered along with adjusting levodopa and other supportive medications.

Consensus regarding a dose increase to 100 mg/day was achieved for cases in which other medications cannot control symptoms, and increasing doses of these medications is difficult, as well as for patients with non-motor symptoms that can negatively affect ADL. Because 50 mg dose of safinamide can almost inhibit MAO-B [26], we believe that the efficacy of 100 mg dose may be explained by the non-dopaminergic actions of safinamide. The results of post hoc analyses of two phase 3 studies showed the efficacy of safinamide 100 mg/day on pain and depressive symptoms [5, 6], which is consistent with our results. Agreement for requiring dose reduction or discontinuation was made in the case of the onset of drug-related AEs, the requirement for anti-depressants, and insufficient improvement of PD-related symptoms.

From our results, the patients for whom safinamide should be administered after carefully balancing risks and benefits were those with a history of hallucinations, high blood pressure variability, and concerns of impulse control disorders (ICDs). These results are similar to the clinical experiences of other MAO-B inhibitors. Other than dyskinesia, common AEs associated with safinamide were reported as visual hallucinations, falls, and constipation in Japanese clinical trials [20, 27]. However, the difference in the risk of AEs between safinamide and other MAO-B inhibitors remains unknown.

Regarding the effects of safinamide on ICD, a similar action to amantadine has been suggested. Even though several meta-analyses, which included cross-sectional studies, showed the use of amantadine as a factor related to ICD [28], several small-scale prospective interventional studies showed that pathological gambling and punding were improved by amantadine [29, 30]. Amantadine enhances dopamine release and acts as an N-methyl-D-aspartate receptor antagonist, and its anti-glutamatergic action may show benefits in ICD.

Reports of safinamide use in elderly patients are limited. Although most patients with PD in Japan are at least 75 years old, the mean age of patients in two clinical studies in Japan was 67–69 years [20, 27]. The results of the SYNAPSES study showed that there were no major differences in occurrence and severity of AEs between patients aged ≤ 75 years and >75 years, but the incidence of serious AEs was 13.6% in patients aged > 75 years vs 7.7% in patients aged ≤ 75 years. The incidence of hallucination was also higher in patients aged > 75 years; however, the incidence of dyskinesia was relatively low in these patients [13]. As there is no clear dosing recommendation in elderly patients, the initial safinamide dose should be the same as that in non-elderly patients (50 mg/day); however, careful observation for AEs, including hallucinations, is required. If there are no tolerability concerns, a dose increase to 100 mg/day is possible in elderly patients. Although the evidence on the efficacy and safety of safinamide in patients with cognitive impairment is limited, a significant improvement in cognitive function (Frontal Assessment Battery, Stroop Word Color Test) has been reported [31]. Furthermore, a retrospective analysis for urinary symptoms showed that Scales for Outcomes in Autonomic Dysfunction improved after safinamide treatment [32]. Such clinical effects on cognition and urinary symptoms may be beneficial for elderly patients.

Many responses in this study indicated that 2 to 3 months are appropriate to evaluate efficacy (87.0%) and the onset of dyskinesia (91.3%). While significant prolongation of ON time at 2 and 4 weeks was shown in global and Japanese phase 3 studies, respectively [12, 20], an improvement over time in the Unified Parkinson's Disease Rating Scale Part III score is evident from clinical study data. This may explain the many responses in favor of evaluating efficacy at 2 to 3 months. Furthermore, it is reasonable to take time to evaluate the onset of dyskinesia because this symptom is known to fluctuate. In contrast, regarding the decision to continue safinamide at the occurrence of AEs, many responses (89.1%) stated that it was appropriate to discontinue or decrease the treatment dose immediately or within 1 month after the AE onset. AEs, including hallucinations, require immediate action, explaining why many responses supported a shorter period.

There were few opinions (39.1%) regarding a lack of relationship between the temporal administration of medication and efficacy, and the agreement consensus was that the timing of taking medication should be tailored to the targeted symptoms: medication after dinner for nocturnal symptoms; medication before going to bed for morning-off; and medication after breakfast for daytime symptoms. It is difficult to explain this based on the mechanism of action because the main effect of safinamide is MAO-B inhibition. This result is considered to be related to the placebo effect on the specific symptoms after administration.

The Delphi approach is one of the most reliable methods for forming a consensus, but difficulties in setting appropriate questions and bias by alternative responses are evident. Furthermore, the definition of PD-related pain was not expanded upon, and so it is not possible to accurately determine whether the reported pain was specifically related to PD. This study was limited because the consensus results were derived from a cross-disciplinary survey in only one country. Additionally, there are variabilities in doctors' experiences with the use of safinamide.

## 5. Conclusions

In this study, the determination of the optimal patient type for the use of safinamide was based on motor/non-motor symptoms and treatment history. In addition, valuable opinions regarding the criteria for dose increase or discontinuation and clinical action to take at the onset of dyskinesia were collected. The results of this study provide useful information as a clinical practice guide for safinamide in patients with wearing-off.

## Figures and Tables

**Table 1:** Consensus of clinically important indicators or methods for the use of safinamide in each scenario.

Rank	Cases or methods that achieved consensus agreement ((%) of agreement)
	Optimal patient profiles: selection of safinamide based on history of receiving other MAO-B inhibitors (Q4)
1	Can become a treatment option in MAO-B inhibitor-naïve patients (97.8%)
2	Can become a treatment option in patients in whom the efficacy of other MAO-B inhibitors was not sufficient in the past (95.7%)
3	Can become a treatment option in patients who previously experienced adverse events related to other MAO-B inhibitors (91.3%)

	Treatment methods: patients for whom safinamide should be administered after carefully balancing risks and benefits of treatment (Q7)
1	Experienced hallucinations or drug-induced hallucinations (93.5%)
2	Concerns of impulse control disorder (87.0%)
3	High blood pressure variability (82.6%)

	Treatment methods: cases in which dose increase to 100 mg/day is recommended (Q10)
1	Insufficient improvement of symptoms and difficulty in dose increase of other medication (97.8%)
2	Insufficient effect of another MAO-B inhibitor at the approved dose (97.8%)
3	Non-motor symptoms that affect daily life (93.5%)

	Treatment methods: cases in which dose reduction or discontinuation is required (Q11)
1	No improvement in symptoms (97.8%)
2	Occurrence of adverse reactions (hallucination, sleepiness, orthostatic hypotension) (95.7%)
3	Requirement for use of anti-depressants (95.7%)

	Treatment methods: safinamide use in elderly (aged ≥ 75 years) patients with Parkinson's disease (Q8)
1	Adopt the same usage and cautions as in non-elderly patients (100.0%)
2	Be careful of the occurrence of psychiatric symptoms, hallucination, or visual hallucination (97.8%)
3	Confirm the degree of hepatic toxicity (95.7%)
4	Dose increase is possible if there is no concern of tolerability (95.7%)
5	Initial dose should be 50 mg/day (93.5%)

Only cases that met consensus (≥80%) were extracted. MAO: monoamine oxidase.

## Data Availability

Due to restrictions by Eisai Co., Ltd., individual data from this research may not be made publicly available. However, the datasets, including the study protocol supporting results reported in this article, can be made available from reasonable request to the corresponding author.

## Supplementary Materials

Supplementary Materials Supplementary Table 1. Levels of evidence. Supplementary Table 2. Studies of safinamide by clinical evidence level (up to May 2021). Supplementary Table 3. Demographics of panelists in the Delphi review. Supplementary Table 4. Questionnaires and responses.
