# Supplementary material for: Collective Expert Perspectives on the Use of Safinamide as Adjunctive Therapy for Parkinson's Disease: Online-Based Delphi Survey
Source: Parkinsons Dis. 2022 Jul 15;2022:3203212. doi: 10.1155/2022/3203212 (PMC9307399; doi:10.1155/2022/3203212)
Supplement: Supplementary Materials — Supplementary Table 1. Levels of evidence. Supplementary Table 2. Studies of safinamide by clinical evidence level (up to May 2021). Supplementary Table 3. Demographics of panelists in the Delphi review. Supplementary Table 4. Questionnaires and responses. [file 3203212.f1.docx]

Supplementary Table 1: Levels of evidence

| **Level*** | **Contents** | **Number of articles** |
| --- | --- | --- |
| 1 | Systematic review, meta-analysis | 8 |
| 2 | Randomized controlled trial, observational study in which a drastic effect or adverse event was observed | 7 |
| 3 | Non-randomized comparison study, *post hoc* analysis of a randomized controlled trial | 11 |
| 4 | Case report (observational study without control group), case-control study, database study, etc. | 22 |
| 5 | Expert’s opinion | 2 |

*Level may be changed because of the quality of the study, lack of accuracy, lack of immediacy (inconsistency between population, intervention, control, and outcomes [PICO] criteria and research question), inconsistency between studies, small absolute effect size (graded down), and large effect size (graded up).

OCEBM Levels of Evidence Working Group “The Oxford 2011 Levels of Evidence” Oxford Centre for Evidence-Based Medicine. http://www.cebm.net/index.aspx?o=5653 (modified)

Supplementary Table 2: Studies of safinamide by clinical evidence level (up to May 2021)

| **First Author_Year_Journal** | **Evidence level** | **Title** |
| --- | --- | --- |
| Binde CD_2020_Eur J Clin Pharmacol | 1 | Comparative effectiveness of dopamine agonists and monoamine oxidase type-B inhibitors for Parkinson’s disease: a multiple treatment comparison meta-analysis |
| Abdelalem Aziz Ahmed M_2019_F1000Res | 1 | A systematic review and meta-analysis of safety and efficacy of safinamide for motor fluctuations in patients with Parkinson’s disease |
| Binde CD_2018_Br J Clin Pharmacol | 1 | A multiple treatment comparison meta-analysis of monoamine oxidase type B inhibitors for Parkinson’s disease |
| Qureshi AR_2018_Neuroepidemiology | 1 | Comprehensive examination of therapies for pain in Parkinson’s disease: a systematic review and meta-analysis |
| Fabbri M_2015_Neurodegener Dis Manag | 1 | Clinical pharmacology review of safinamide for the treatment of Parkinson’s disease |
| Huang YH_2021_Ther Adv Psychopharmacol | 1 | The effect of monoamine oxidase-B inhibitors on the alleviation of depressive symptoms in Parkinson’s disease: meta-analysis of randomized controlled trials |
| Giossi R_2021_Clin Drug Investig | 1 | Overall efficacy and safety of safinamide in Parkinson’s disease: a systematic review and a meta-analysis |
| Fox SH_2018_Mov Disord | 1 | International Parkinson and movement disorder society evidence‐based medicine review: Update on treatments for the motor symptoms of Parkinson’s disease |
| Hattori N_2020_Parkinsonism Relat Disord | 2 | Efficacy and safety of safinamide as an add-on therapy to L-DOPA for patients with Parkinson’s disease: A randomized, double-blind, placebo-controlled, phase II/III study |
| Schapira AH_2017_JAMA Neurol | 2 | Assessment of safety and efficacy of safinamide as a levodopa adjunct in patients with Parkinson disease and motor fluctuations: a randomized clinical trial |
| Borgohain R_2014_Mov Disord | 2 | Randomized trial of safinamide add-on to levodopa in Parkinson’s disease with motor fluctuations |
| Marquet A_2012_Clin Pharmacol Ther | 2 | The effect of safinamide, a novel drug for Parkinson’s disease, on pressor response to oral tyramine: a randomized, double-blind, clinical trial |
| Stocchi F_2012_Mov Disord | 2 | A randomized, double-blind, placebo-controlled trial of safinamide as add-on therapy in early Parkinson’s disease patients |
| Di Stefano AF_2011_Naunyn Schmiedebergs Arch Pharmacol | 2 | Pressor response to oral tyramine during co-administration with safinamide in healthy volunteers |
| Stocchi F_2004_Neurology | 2 | Improvement of motor function in early Parkinson disease by safinamide |
| Borgohain R_2014_Mov Disord | 3 | Two-year, randomized, controlled study of safinamide as add-on to levodopa in mid to late Parkinson’s disease |
| Cattaneo C_2020_J Parkinsons Dis | 3 | Long-term efficacy of safinamide on symptoms severity and quality of life in fluctuating Parkinson’s disease patients |
| Cattaneo C_2018_Adv Ther | 3 | Long-term efficacy of safinamide on Parkinson’s Disease chronic pain |
| Cattaneo C_2017_J Parkinsons Dis | 3 | Long-term effects of safinamide on mood fluctuations in Parkinson’s disease |
| Cattaneo C_2017_J Parkinsons Dis | 3 | Effects of safinamide on pain in fluctuating Parkinson’s disease patients: a post-hoc analysis |
| Cattaneo C_2016_J Parkinsons Dis | 3 | Safinamide as add-on therapy to levodopa in mid- to late-stage Parkinson’s disease fluctuating patients: post hoc analyses of Studies 016 and SETTLE |
| Cattaneo C_2015_J Parkinsons Dis | 3 | Long-Term effects of safinamide on dyskinesia in mid- to late-stage Parkinson’s disease: a post-hoc analysis |
| Schapira AH_2013_Eur J Neurol | 3 | Long-term efficacy and safety of safinamide as add-on therapy in early Parkinson’s disease |
| Müller T_2017_Clin Neuropharmacol | 3 | Determination of monoamine oxidase A and B activity in long-term treated patients with Parkinson disease |
| Stocchi F_2006_Neurology | 3 | Symptom relief in Parkinson disease by safinamide: Biochemical and clinical evidence of efficacy beyond MAO-B inhibition |
| Cattaneo C_2003_Clin Neuropharmacol | 3 | Pressor response to intravenous tyramine in healthy subjects after safinamide, a novel neuroprotectant with selective, reversible monoamine oxidase B inhibition |
| Martí-Andrés G_2019_Brain Sci | 4 | Safinamide in clinical practice: a Spanish multicenter cohort study |
| Liguori C_2018_Parkinsonism Relat Disord | 4 | Safinamide effect on sleep disturbances and daytime sleepiness in motor fluctuating Parkinson’s disease patients: A validated questionnaires-controlled study |
| Lo Monaco MR_2020_Aging Clin Exp Res | 4 | Safinamide as an adjunct therapy in older patients with Parkinson’s disease: a retrospective study |
| Gómez-López A_2021_Brain Sci | 4 | SURINPARK: Safinamide for Urinary Symptoms in Parkinson’s Disease |
| Stocchi F_2020_Eur J Neurol | 4 | Overnight switch from rasagiline to safinamide in Parkinson's disease patients with motor fluctuations: A tolerability and safety study |
| Rinaldi D_2020_Aging Clin Exp Res | 4 | The tolerability, safety and efficacy of safinamide in elderly Parkinson’s disease patients: a retrospective study |
| Tsuboi Y_2020_J Neurol Sci | 4 | Long-term safety and efficacy of safinamide as add-on therapy in levodopa-treated Japanese patients with Parkinson’s disease with wearing-off: Results of an open-label study |
| Geroin C_2020_J Neural Transm (Vienna) | 4 | Effects of safinamide on pain in Parkinson’s disease with motor fluctuations: an exploratory study |
| Guerra A_2019_Brain Stimul | 4 | Abnormal cortical facilitation and L-dopa-induced dyskinesia in Parkinson’s disease |
| Avila A_2019_Acta Neurol Scand | 4 | Rasagiline and safinamide as a dopamine-sparing therapy for Parkinson’s disease |
| Bianchi MLE_2019_Neurol Sci | 4 | Efficacy of safinamide on non-motor symptoms in a cohort of patients affected by idiopathic Parkinson’s disease |
| Mancini F_2018_Neurol Sci | 4 | Real life evaluation of safinamide effectiveness in Parkinson’s disease |
| Loprete L_2016_Pharmacol Res Perspect | 4 | Population pharmacokinetic and pharmacodynamic analyses of safinamide in subjects with Parkinson’s disease |
| Seithel-Keuth A_2013_Clin Pharmacol Drug Dev | 4 | Absolute bioavailability and effect of food on the disposition of safinamide immediate release tablets in healthy adult subjects |
| Marzo A_2004_Pharmacol Res | 4 | Pharmacokinetics and pharmacodynamics of safinamide, a neuroprotectant with antiparkinsonian and anticonvulsant activity |
| Abbruzzese G_2020_J Parkinson Dis | 4 | A European observational study to evaluate the safety and the effectiveness of safinamide in routine clinical practice: the SYNAPSES trial |
| Rinaldi D_2020_J Neur Transm | 4 | Safinamide improves executive functions in fluctuating Parkinson’s disease patients: an exploratory study |
| Peña E_2021_Brain Sci | 4 | Impact of SAfinamide on Depressive Symptoms in Parkinson’s Disease Patients (SADness-PD Study): a multicenter retrospective study |
| Santos García D_2021_Brain Sci | 4 | Safinamide improves non-motor symptoms burden in Parkinson’s disease: an open-label prospective study |
| Puy-Núñez A_2020_Neurologia | 4 | Hypersexuality associated with safinamide |
| Liguori C_2018_Sleep Med | 4 | Effective treatment of restless legs syndrome by safinamide in Parkinson’s disease patients |
| Jiménez-Jiménez FJ_2017_J Clin Psychopharmacol | 4 | Hypersexuality possibly associated with safinamide |
| Fackrell R_2018_Neurodegener Dis Manag | 5 | Noninvasive options for ‘wearing-off’ in Parkinson’s disease: a clinical consensus from a panel of UK Parkinson’s disease specialists |
| Müller T_2017_Clin Pharmacokinet | 5 | Clinical pharmacokinetics and pharmacodynamics of safinamide |

Supplementary Table 3: Demographics of panelists in the Delphi review

| **Characteristic** | **First-round survey**  **(*n* = 150)** | **Second-round survey**  **(*n* = 46)** |
| --- | --- | --- |
| Sex (male), % | 92.7 | 93.5 |
| Age, years | 51.2 ± 10.7 | 52.0 ± 9.8 |
| Department, % |  |  |
| Neurology | 76.7 | 80.4 |
| Neurosurgery | 17.3 | 19.6 |
| Other | 6.0 | 0.0 |
| Clinical practice experience in treating Parkinson’s disease, years | 22.3 ± 9.4 | 22.9 ± 8.6 |
| Number of patients with Parkinson’s disease for whom clinical care was provided | 65.9 ± 80.3 | 116.6 ± 131.5 |
| Number of patients prescribed safinamide | 5.2 ± 7.2 | 14.0 ± 18.1 |

Data are mean ± standard deviation, unless otherwise indicated.

Supplementary Table 4: Questionnaires and responses

|  | **Responses (%)** | | | | | **Level of consensus** |
| --- | --- | --- | --- | --- | --- | --- |
|  | **Disagree**  **(1)** | **Moderately disagree**  **(2)** | **Moderately agree**  **(3)** | **Agree**  **(4)** | **Agreement rate**  **(3 and 4)** |  |
| **Q1. Optimal patient type based on motor symptoms related to wearing-off** |  |  |  |  |  |  |
| Patients with bradykinesia | 0.0 | 0.0 | 37.0 | 63.0 | 100.0 | Agreement consensus |
| Patients with tremor | 0.0 | 26.1 | 56.5 | 17.4 | 73.9 | Agreement nearing consensus |
| Patients with rigidity | 0.0 | 4.3 | 69.6 | 26.1 | 95.7 | Agreement consensus |
| Patients with axial symptoms | 0.0 | 21.7 | 54.3 | 23.9 | 78.3 | Agreement nearing consensus |
| Patients with gait disorder (including freezing phenomenon) | 0.0 | 10.9 | 60.9 | 28.3 | 89.1 | Agreement consensus |
| Patients with speech problem | 0.0 | 19.6 | 63.0 | 17.4 | 80.4 | Agreement consensus |
| Patients with masked face | 4.3 | 15.2 | 65.2 | 15.2 | 80.4 | Agreement consensus |
| **Q2. Optimal patient type based on motor complications** |  |  |  |  |  |  |
| Patients with wearing-off | 0.0 | 0.0 | 19.6 | 80.4 | 100.0 | Agreement consensus |
| Patients with disability in daily life due to wearing-off (ex. OFF-time per day ≥2 hours) | 0.0 | 0.0 | 30.4 | 69.6 | 100.0 | Agreement consensus |
| Patients with morning-off | 0.0 | 4.3 | 34.8 | 60.9 | 95.7 | Agreement consensus |
| Patients with dyskinesia | 4.3 | 23.9 | 58.7 | 13.0 | 71.7 | Agreement nearing consensus |
| **Q3. Cases of using safinamide in patients with wearing-off** |  |  |  |  |  |  |
| Insufficient symptom improvement while taking levodopa 3 times a day | 0.0 | 6.5 | 39.1 | 54.3 | 93.5 | Agreement consensus |
| Insufficient symptom improvement while taking levodopa 4 to 5 times a day | 0.0 | 0.0 | 32.6 | 67.4 | 100.0 | Agreement consensus |
| Insufficient symptom improvement while taking levodopa and a dopamine agonist(s) | 0.0 | 4.3 | 32.6 | 63.0 | 95.7 | Agreement consensus |
| Insufficient symptom improvement while taking one additional medication (COMT inhibitor, zonisamide, istradefylline, etc.) concomitantly with levodopa and a dopamine agonist(s) | 0.0 | 4.3 | 39.1 | 56.5 | 95.7 | Agreement consensus |
| Insufficient symptom improvement while receiving DBS | 0.0 | 10.9 | 63.0 | 26.1 | 89.1 | Agreement consensus |
| Insufficient symptom improvement while receiving LCIG | 2.2 | 13.0 | 67.4 | 17.4 | 84.8 | Agreement consensus |
| **Q4. Selection of safinamide based on history of receiving other MAO-B inhibitors** |  |  |  |  |  |  |
| Can become a treatment option in MAO-B inhibitor-naïve patients | 0.0 | 2.2 | 54.3 | 43.5 | 97.8 | Agreement consensus |
| Can become a treatment option in patients who previously experienced adverse events related to other MAO-B inhibitor(s) | 0.0 | 8.7 | 47.8 | 43.5 | 91.3 | Agreement consensus |
| Can become a treatment option in patients in whom the efficacy of other MAO-B inhibitor(s) was not sufficient in the past | 0.0 | 4.3 | 52.2 | 43.5 | 95.7 | Agreement consensus |
| **Q5. Action taken for troublesome dyskinesia during safinamide use** |  |  |  |  |  |  |
| Dose reduction or discontinuation of safinamide | 2.2 | 6.5 | 58.7 | 32.6 | 91.3 | Agreement consensus |
| Adjusting the dose or frequency of levodopa while continuing safinamide | 0.0 | 8.7 | 73.9 | 17.4 | 91.3 | Agreement consensus |
| Dose reduction of other anti-PD medication(s) (other than levodopa) while continuing safinamide | 0.0 | 4.3 | 82.6 | 13.0 | 95.7 | Agreement consensus |
| Continuing observation without dose change of any anti-PD medications, including safinamide | 6.5 | 65.2 | 23.9 | 4.3 | 28.3 | Disagreement nearing consensus |
| Dose increase of safinamide | 21.7 | 50.0 | 21.7 | 6.5 | 28.3 | Disagreement nearing consensus |
| **Q6. Action taken in using safinamide in patients with non-troublesome dyskinesia** |  |  |  |  |  |  |
| Dose reduction or discontinuation of other anti-PD medication(s) other than levodopa | 6.5 | 17.4 | 67.4 | 8.7 | 76.1 | Agreement nearing consensus |
| Reduction of levodopa daily dose and supplementation with safinamide for the resulting shortage | 4.3 | 21.7 | 67.4 | 6.5 | 73.9 | Agreement nearing consensus |
| Adding safinamide without adjusting the dose or any other anti-PD medications | 4.3 | 34.8 | 54.3 | 6.5 | 60.9 | Agreement nearing consensus |
| **Q7. Patient type for whom safinamide should be administered after carefully balancing risks and benefits of treatment (other than those with dyskinesia)** |  |  |  |  |  |  |
| Patients who have experienced drug-induced hallucinations or visual hallucinations | 0.0 | 6.5 | 80.4 | 13.0 | 93.5 | Agreement consensus |
| Patients with high blood pressure variability (e.g., concerns of asymptomatic orthostatic hypotension) | 0.0 | 17.4 | 65.2 | 17.4 | 82.6 | Agreement consensus |
| Patients with depressive symptoms | 4.3 | 47.8 | 34.8 | 13.0 | 47.8 | No consensus |
| Patients engaged in high-risk work, including car driving or machine operating | 0.0 | 26.1 | 60.9 | 13.0 | 73.9 | Agreement nearing consensus |
| Patients with concerns of impulse control disorder | 0.0 | 13.0 | 78.3 | 8.7 | 87.0 | Agreement consensus |
| **Q8. Treatment management when using safinamide in elderly patients with PD (≥75 years)** |  |  |  |  |  |  |
| Initial dose of 50 mg/day (low dose) is preferable | 2.2 | 4.3 | 60.9 | 32.6 | 93.5 | Agreement consensus |
| Careful observation for the occurrence of psychiatric symptoms, hallucinations, or visual hallucinations is needed | 0.0 | 2.2 | 60.9 | 37.0 | 97.8 | Agreement consensus |
| Degree of hepatic impairment should be checked | 0.0 | 4.3 | 78.3 | 17.4 | 95.7 | Agreement consensus |
| Dose increase is allowed when there are no tolerability concerns | 2.2 | 2.2 | 73.9 | 21.7 | 95.7 | Agreement consensus |
| The same usage and cautions for non-elderly patients can be adopted for elderly patients | 0.0 | 0.0 | 78.3 | 21.7 | 100.0 | Agreement consensus |
| **Q9. Optimal patient type based on non-motor symptoms associated with wearing-off** |  |  |  |  |  |  |
| Patients with depressive symptoms or apathy associated with PD | 0.0 | 6.5 | 69.6 | 23.9 | 93.5 | Agreement consensus |
| Patients with PD-related pain | 0.0 | 2.2 | 78.3 | 19.6 | 97.8 | Agreement consensus |
| Patients with strong daytime somnolence | 2.2 | 17.4 | 69.6 | 10.9 | 80.4 | Agreement consensus |
| Patients with sleep disorder (sleep-onset insomnia or wake after sleep onset) | 2.2 | 21.7 | 69.6 | 6.5 | 76.1 | Agreement nearing consensus |
| Patients with a urinary problem (including nocturia) | 0.0 | 34.8 | 58.7 | 6.5 | 65.2 | Agreement nearing consensus |
| Patients with cognitive impairment | 2.2 | 26.1 | 69.6 | 2.2 | 71.7 | Agreement nearing consensus |
| **Q10. Cases in which dose increase of safinamide to 100 mg/day (high dose) is recommended** |  |  |  |  |  |  |
| Insufficient improvement of symptoms and difficulty in dose increase of other anti-PD medications (levodopa and dopamine agonist, etc.) | 0.0 | 2.2 | 67.4 | 30.4 | 97.8 | Agreement consensus |
| Having non-motor symptoms that affect daily life | 0.0 | 6.5 | 69.6 | 23.9 | 93.5 | Agreement consensus |
| Occurrence of troublesome dyskinesia | 8.7 | 47.8 | 30.4 | 13.0 | 43.5 | No consensus |
| Occurrence of non-troublesome dyskinesia | 4.3 | 23.9 | 67.4 | 4.3 | 71.7 | Agreement nearing consensus |
| Insufficient effect with approved dose of another MAO-B inhibitors | 2.2 | 0.0 | 71.7 | 26.1 | 97.8 | Agreement consensus |
| **Q11. Cases in which dose reduction or discontinuation of safinamide is required (other than the occurrence of dyskinesia)** |  |  |  |  |  |  |
| Occurrence of adverse events (hallucination, somnolence, orthostatic hypotension, etc.) | 0.0 | 4.3 | 65.2 | 30.4 | 95.7 | Agreement consensus |
| Onset of depressive symptom and requirement for anti-depressants | 0.0 | 4.3 | 78.3 | 17.4 | 95.7 | Agreement consensus |
| No improvement of symptoms | 0.0 | 2.2 | 65.2 | 32.6 | 97.8 | Agreement consensus |
| **Q13. Timing to consider discontinuation or dose reduction of safinamide** |  |  |  |  |  |  |
| Immediately to within 1 month after the occurrence of dyskinesia | 2.2 | 15.2 | 76.1 | 6.5 | 82.6 | Agreement consensus |
| Two to three months after the occurrence of dyskinesia | 2.2 | 6.5 | 82.6 | 8.7 | 91.3 | Agreement consensus |
| Immediately to within 1 month after the occurrence of other adverse events (hallucination, somnolence, etc.) | 0.0 | 10.9 | 80.4 | 8.7 | 89.1 | Agreement consensus |
| Two to three months after the occurrence of other adverse events (hallucination, somnolence, etc.) | 2.2 | 15.2 | 73.9 | 8.7 | 82.6 | Agreement consensus |
| **Q14. Timing of safinamide administration** |  |  |  |  |  |  |
| After dinner or before going to bed, if early morning-off symptoms | 0.0 | 8.7 | 76.1 | 15.2 | 91.3 | Agreement consensus |
| After dinner or before going to bed if strong nocturnal symptoms | 0.0 | 10.9 | 73.9 | 15.2 | 89.1 | Agreement consensus |
| After breakfast, if expecting improvement of symptoms during daytime | 0.0 | 8.7 | 76.1 | 15.2 | 91.3 | Agreement consensus |
| No relationship between timing of administration and effect | 0.0 | 60.9 | 30.4 | 8.7 | 39.1 | Disagreement nearing consensus |
| **Q15. Concomitant use of safinamide with other anti-PD medications or physiotherapy** |  |  |  |  |  |  |
| Concomitant use of safinamide with a COMT inhibitor is useful | 0.0 | 13.0 | 69.6 | 17.4 | 87.0 | Agreement consensus |
| Concomitant use of safinamide with a dopamine agonist transdermal patch is useful | 0.0 | 17.4 | 54.3 | 28.3 | 82.6 | Agreement consensus |
| Concomitant use of safinamide with an oral dopamine agonist is useful | 0.0 | 15.2 | 63.0 | 21.7 | 84.8 | Agreement consensus |
| Safinamide therapy is useful during physiotherapy or occupational therapy | 0.0 | 13.0 | 63.0 | 23.9 | 87.0 | Agreement consensus |

| **Q12. Timing of efficacy evaluation of safinamide** | View as important |
| --- | --- |
| One month after the start of administration | 13.0 |
| Two to three months after the start of administration | 87.0 |

The level of consensus categories are as follows: agreement consensus, 80% to 100%; agreement nearing consensus, 60% to 79%; no consensus, 41% to 59%; disagreement nearing consensus, 21% to 40%; disagreement consensus, 0 to 20%.

PD: Parkinson’s disease, COMT: catechol-O-methyltransferase, DBS: deep brain stimulation, LCIG: levodopa continuous intestinal gel, MAO: monoamine oxidase.
